# Extended Theoretical Framework of Parental Internet Mediation: Use of Multiple Theoretical Stances for Understanding Socio-Ecological Predictors

**DOI:** 10.3389/fpsyg.2021.620838

**Published:** 2021-06-09

**Authors:** Sarosh Iqbal, Rubeena Zakar, Florian Fischer

**Affiliations:** ^1^Institute of Social and Cultural Studies, University of the Punjab, Lahore, Pakistan; ^2^Department of Public Health, Institute of Social and Cultural Studies, University of the Punjab, Lahore, Pakistan; ^3^Institute of Public Health, Charité – Universitätsmedizin Berlin, Berlin, Germany; ^4^Institute of Gerontological Health Services and Nursing Research, Ravensburg-Weingarten University of Applied Sciences, Weingarten, Germany

**Keywords:** digitalization, internet addiction, theory, resilience, children, teenager

## Abstract

Digital media is a common phenomenon in contemporary societies. Recognizing the popularity of digital and online devices among the younger generation, the subject of parental internet mediation is of utmost significance for avoiding the adverse effects of digital media on the physical, cognitive, and social wellbeing of youngsters. Taking insights from an informed review of multi-grounded theories, we propose an extended framework of socio-ecological predictors concerning parental internet mediation. This contribution offers an innovative methodological and analytical perspective to consider both psychological and behavioral predictors for promoting resilience. This review acknowledged resilience as a strength-based measure to ensure online safety among young individuals. This review suggests that an integrated socio-ecological approach is critical to formulating the basis for a theoretical framework to fully comprehend the socio-ecological predictors of parental internet mediation.

## Introduction

Parental mediation in internet use of young children, teenagers, and adolescents is widespread in both developed and developing countries. It is one of the critical agendas of contemporary societies because almost all young individuals, growing up in the age of digitalization, use media and the internet in their daily routine (Hassan et al., 2020). The relationship of youngsters with digital media is diverse, highlighting multiple challenges in a varied sociocultural context (Green et al., 2020). The harmful effects of digital media have been discussed in recent years, particularly focusing on the physical and psychological health of the younger generation, including children and teenagers (Ferrara et al., 2017; Bruggeman et al., 2019; Biernesser et al., 2020; Hassan et al., 2020; Twenge and Martin, 2020). Large evidence pointing to issues such as internet addiction (Kawabe et al., 2016), cyberbullying and cybervictimization (John et al., 2018; Lin et al., 2020), and adverse health effects of online gaming (Paulus et al., 2018; Stenseng et al., 2020) is available. Predominantly, the outcomes are related not only to cognitive and mental health and sociability indicators (Bilgrami et al., 2017; Green et al., 2020) but also to self-inflicted violence (Deslandes and Coutinho, 2020), sleep habits, and quality of life (Kawabe et al., 2019).

To reduce the adverse effects of digital media on the physical, cognitive, and social wellbeing of children and teenagers, parental mediation is expected to be a successful approach. Parental mediation is a multi-pronged concept and facilitates in building and enhancing essential digital skills (Livingstone, 2008; Lin et al., 2019; Green et al., 2020). The concept of parental mediation is mainly situated within the media effects paradigm, which is primarily rooted in communication and deals with social, psychological, and developmental perspectives (Mendoza, 2009; Clark, 2011; Livingstone et al., 2015a). Reciprocal relationship between parents and the younger generation, their social environment, and psychological needs is of utmost importance in this regard (Clark, 2011; Green et al., 2020). The discourse of parental mediation explores how parents interact, regulate, or manage the use of media or the internet, particularly in mitigating negative effects on the physical, psychological, and emotional health and sociability of children/teenagers (Mendoza, 2009; Green et al., 2020).

Parents are the main socializing agents and gatekeepers for the younger generation to access media and digital devices. They ensure the adequate use of media regarding content and time by their children. In this study, the younger generation is referred to as children, teenagers, and adolescents. Research on parental mediation encompasses all of these age groups. This is due to the fact that childhood, teenage, and adolescence all are transitional phases of human life, where young individuals experience new things, reject normative ideas, and prefer to adopt unique lifestyles (Flanagan et al., 2015). Though, there is a difference among the nomenclature and age groups due to the transition from childhood to adolescence, where children include both preschool and school-age children, teenagers (13–19 years), and adolescents [10–19 years; Flanagan et al., 2015; Aierbe et al., 2019; WHO (World Health Organization), n.d.]. Nonetheless, Havighurst (1972) labeled children, teenagers, and adolescents as a unitary group in view of their developmental tasks. Further, these are the most common age groups used in the literature concerning parental mediation because parental control plays a decisive role in the regulation of their behavior. Furthermore, these groups are most vulnerable to the risks associated with both media and internet due to the lack of e-maturity, more close contact with peers, inadequate capacity for self-regulation, and self-efficacy to cope with risks (O’Keeffe and Clarke-Pearson, 2011; Aierbe et al., 2019). For that reason, they require parental attention and mediation.

Based on the abovementioned context, this review acknowledges the need for an overarching framework of parental internet mediation in the context of a digital media-rich environment. Therefore, it aims to seek guidance from the review of multi-grounded theories to enrich the sociological inquiry and design an extended framework for the socio-ecological predictors of parental internet mediation. This review is structured into four main sections. Section 1 describes the introduction and context of the study, followed by the objective and structure of the study. Section 2 highlights the contribution of the existing literature to parental internet mediation and explains the particular theoretical assumptions of multi-grounded theories. This section critically debates and identifies key predictors of parental internet mediation, which impact at various levels (the level of children/teenagers, parental level, and contextual level). Section 3 synthesizes, summarizes, and proposes an extended theoretical framework for the socio-ecological predictors and motivating factors of parental internet mediation, followed by the conclusion in Section 4.

## Predictors of Parental Internet Mediation

Digital media and technology lead to both advantages and challenges in contemporary societies. This contribution focuses on parental internet mediation, recognizing the popularity of digital and online devices among the younger generation. This article discourses on various predictors of parental internet mediation, using relevant theoretical stances to comprehensively extend the existing framework with a socio-ecological approach. Although excessive use of the internet is one of the significant predictors of parental internet mediation, nonetheless, there are other multiple contributing and contextual factors, which are essential to be considered, enhancing digital literacy and empowering the younger generation to manage risks (Lin et al., 2019).

Various strands correspond to comprehend the framework and ideologies of parental internet mediation and their related factors. At the very onset, the academic discourse of parental mediation emerged from communication and media studies due to the advent of TV and video games. At that time, communication and media experts were concerned to understand the effects of TV and video games on the behaviors of children (Rothfuss-Buerkel and Buerkel, 2001; Livingstone et al., 2015a; Aierbe et al., 2019). However, soon it evolved as an important subject in the field of social psychology to comprehend the motives of parental mediation, in view of an array of digital devices and media-rich environment (Clark, 2011; Iqbal, 2019; Lin et al., 2019; Iqbal et al., 2021). In this context, the parental mediation theory emerged and underwent a critical review over a period of time (Clark, 2011; Iqbal, 2019).

Researchers coined various typologies on parental mediation to address the changing context of media and digital devices. They attempted to understand parental mediation and its related factors to apply various methodological and philosophical assumptions and to share some common questions, nonetheless with different theoretical perspectives (Clark, 2012). The variation in opinions of researchers highlights their own conception of theory and its application according to their own context and interests (Clark, 2012). This argument is well-established by the fact that the word “theory” is a broader and an applied term, which has diverse meanings in various disciplines such as sociology and communication.

In the field of communication, Craig (1999) observed that theory is a radically different conception and discussion of communication problems and practices. Similarly, theory or semantic predicament is an important expression in the lexicon of contemporary sociology, having multiple definitions and concepts (Abend, 2008). Abend (2008) categorized numerous definitions, describing that theory is a “logically connected system of general propositions,” an “explanation of a particular social empirical phenomenon,” an “overall perspective to see and interpret the social world,” an “account of a fundamental normative component,” and the “discussions about the ways by which reality is socially constructed.” Broadly, theory may be comprehended as the explanation and interpretation of a social phenomenon within a social context.

Taking insights from various theories, this contribution is intended to provide some critical aspects for extending the framework of socio-ecological predictors of parental internet mediation. Largely, a theoretical framework is viewed as an organizing structure, which conveys meanings to any situation and creates linkages between predictors (Baldwin et al., 2003). It is observed that social, psychological, and communication researchers used several methodological approaches for understanding the social context and related factors which influence behavior (Clark, 2012). Based on the analysis of the parental internet mediation literature, this study argues to adopt a multi-grounded theoretical framework, accounting for both parental and children level factors. This contribution, an interplay of various theories, is found to be interesting and relevant for researches to explore various predictors of parental internet mediation, applying both quantitative and qualitative research methods to explain social reality (Iqbal, 2019). In the following sections, we present an overview of multiple theoretical perspectives, followed by a debate along with a comprehensive, proposed, and extended socio-ecological theoretical framework. Keeping in view the objective of this study, four theories have been selected for the academic discourse: the parental mediation theory, the ecological theory of development, the protection motivation theory, and the theory of resilience. These are explained in the following subsections. The parental mediation theory was prioritized, as it is the focus of this study, while the ecological theory of development (Bronfenbrenner, 1979) was chosen by considering the developmental stages of children/teenagers. Furthermore, the protection motivation theory (Norman et al., 2005; Plotnikoff and Trinh, 2010) was used to understand the preference of parents for mediation in view of vulnerability of youngsters to online risks and efficacy of coping with the risks. Finally, the social and ecological theory of resilience (Ungar, 2011) was incorporated to extend the theoretical assumptions of parental internet mediation. In this contribution, resilience is acknowledged as a strength-based measure and positive adjustment against challenges or risks to ensure online safety.

### Parental Mediation Theory

The parental mediation theory was originated to examine the effects of TV on children and teenagers in media and communication. Initially, researchers coined three dimensions of parental mediation, i.e., active, restrictive, and co-view mediation (Nathanson, 1999, 2002; Martins et al., 2015), which were, later on, applied to video games, the internet, and smartphones. St. Peters et al. (1991) expanded the parental mediation theory in four dimensions, which are distinguished according to the type (active vs. regulated) and level (high vs. low) of mediation. High levels of active and regulative mediation are referred to as selective mediation, whereas low levels of both are called as Laisses Faire (or unmediated). Highly active and low regulated mediation is promotive and highly regulated but low active is labeled as restrictive mediation (Wright et al., 1990; St. Peters et al., 1991; Truglio et al., 1996; Livingstone and Helsper, 2008).

In addition, Clark (2011) adopted a critical stance for the parental mediation theory and employed participatory learning as a further dimension of parental mediation, highlighting the role of communication between the parents and their children for being active participants. However, Clark (2011) identified gaps in the existing parental mediation theory in relation to digital and mobile media. Nikken and Jansz (2014) addressed this gap and introduced five dimensions of parental mediation to regulate online behavior in view of digital media: active, restrictive, co-use mediation on access, content, and supervision. Livingstone et al. (2015b) adopted a holistic approach and acknowledged the complexity of online digital and portable devices, specifically for parents to manage. Livingstone et al. (2015b) also categorized five dimensions of parental internet mediation, which are more comprehensive and widely adopted: active co-use or instructive, restrictive, monitoring, technical, and active internet safety mediation. Later, Livingstone et al. (2017) defined two broader dimensions of parental internet mediation, i.e., enabling and restrictive mediation, which provides a reorganization of the dimensions and underlines the interactive nature of mediation. Enabling mediation encompasses active mediation in coupling with safety and technical mediation and monitoring. Although the abovementioned five dimensions are very pertinent in the present digital media-rich environment; however, we believe that Laissez Faire or unmediated dimension is also essential and must be included as the sixth dimension of parental internet mediation, as few parents either mediate less or remain unmediated according to their cultural practices (Iqbal, 2019; Iqbal et al., 2021), which may affect the positive outcomes of children/teenagers.

To sum up, the parental mediation theory has evolved over time with the growth of media and digital technology and classified six key dimensions, which are essential to be considered: active co-use or instructive, restrictive, monitoring, technical and active internet safety mediation, Laissez Faire, or unmediated. Along with the dimensions of parental mediation, several researchers also identified some predictors of parental internet mediation (Clark, 2011; Symons et al., 2017; Clay, 2019; Lin et al., 2019). However, to establish a more thorough understanding and explanation of the socio-ecological predictors of parental internet mediation, the consultation of further theories is required as explained in the following sections.

### Ecological Theory of Development

Clark (2011) found few limitations in the parental mediation theory in terms of its prime focus on negative effects of media on the cognitive development of children, while overlooked processes shaping parental mediation, especially the parent–child relationship. Therefore, Clark (2011) recommended that attention should be paid to the sociology of childhood to delimit the parental mediation research. In this context, the ecological theory of development has been selected for an extended framework. The ecological theory of development by Bronfenbrenner (1979) provides a basis for understanding the environment of children and teenagers in the context of development classifying into the following five integrated systems:

- Microsystem: the relationship between children/teenagers and an immediate environment, such as family and friends.
- Mesosystem: the relationship between two or more microsystems where children/teenagers actively participate, such as at home and school.
- Exosystem: an indirect environment, which has a bearing on children/teenagers, such as the working place of parents.
- Macrosystem: the prevailing sociocultural and economic conditions of society.
- Chronosystem: the system of nested relationships, capturing the lower subsystems over time.

The ecological theory highlights certain critical factors of development, shaping the environment of children and teenagers. Specifically, this theory enables in comprehending the current scenario of media and digital landscape around every child/teenager at the level of the home, school/college, and community. Though this theory was developed prior to the revolution of the internet and digital media as well as their developmental impact on children/teenagers, yet, it is considered as the most comprehensive one, focusing on their immediate, direct, and indirect environments.

Atkin (2001) and Jordan (2004) added that an ecological and overarching perspective, centered on home, is most significant to understand the environment of children/teenagers as digital natives, and their relationships within micro-, meso-, and exo-system. Further, Johnson and Puplampu (2008) acknowledged the role of technology and embedded the concept of ecological techno-subsystem through introducing a new dimension in an immediate environment (microsystem), concerning the interactions of children/teenagers with both living (e.g., peers) and non-living elements of technology (e.g., hardware and digital devices).

Livingstone et al. (2012) also adopted an ecological approach for investigating online-based activities of children/teenagers and parental mediation within the context of social structures of family, community, and culture. They added three categories while investigating parental internet mediation, i.e., individual-level or microsystem (e.g., home), social mediation (e.g., school/college and peers), and national-level or macrosystem (e.g., cultural values, socio-economic conditions, and regulations). In addition, Livingstone et al. (2015b) also refined an ecological and analytical model and identified various factors at the individual, society, and national level, encompassing demographics, online access, activities, and risks of children/teenagers. Thus, previous researchers applied the ecological theory within media and internet-related studies to comprehend the interplay of various factors (Livingstone et al., 2012, 2015b).

Given this context, we included an ecological theoretical perspective to highlight the predictors within the five integrated systems. Since the use of the internet is more personalized and individualized, it is essential to understand the factors concerning the access of children/teenagers. Primarily, the role of the digital environment at home and school/college is most essential to be considered, where children and teenagers have instant access to the internet and internet-connected devices.

**Figure 1:**
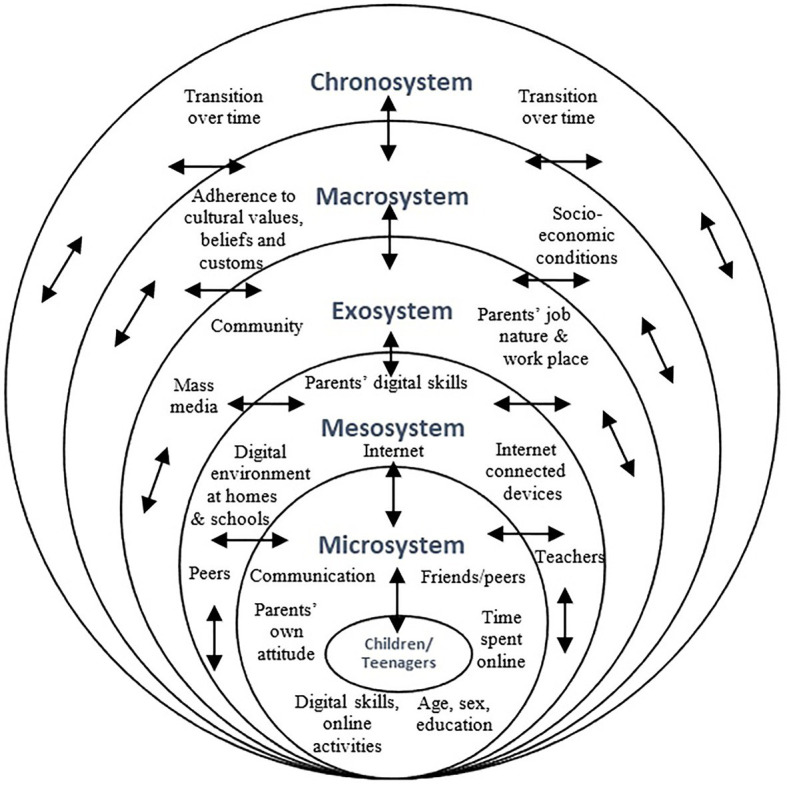
Internet-related ecological system.

In view of a digital media-rich environment, we recommend the following predictors using an ecological approach for an extended parental internet mediation theoretical framework (Figure 1), positioning children/teenagers within their environment and nested within the five key integrated systems. These predictors are proposed at the level of children/teenagers, parents, and contexts.

- The microsystem includes (i) the characteristics of children/teenagers, e.g., age, sex, the level of education, digital skills, types of online activities, and time spent online and (ii) the characteristics concerning their parents, e.g., parental attitude toward the use of internet; level of communication between parent–child/teenager; and the role of peers/friends in the use of internet.
- The mesosystem comprises (i) the characteristics of the digital environment of children/teenagers at home and schools/colleges such as availability of internet (WIFI and mobile data package dongles) and internet-connected devices (computer, laptop, tablets, smartphones, smart TV, audio devices, and video game devices); (ii) parental digital skills; and (iii) the role of teachers and peers/friends.
- The exosystem includes (i) the characteristics concerning their parents, e.g., parental job nature and workplace and (ii) contextual factors, e.g., the role of mass media and community support.
- The macrosystem consists of adherence to prevailing cultural values, beliefs, and customs as well as socio-economic conditions, as contextual factors.
- The chronosystem highlights the transition over time.

It is envisaged that these predictors altogether at various levels influence the engagement of children/teenagers with digital devices as well as parental preferences for various dimensions of internet mediation in interactive and multilateral ways.

### Protection Motivation Theory

Taking the frame of reference of parents, the protection motivation theory provides another perspective to explore the factors of parental internet mediation. This theory postulates that intention of an individual to engage in protection behaviors is based on his/her understanding of the adversity, i.e., fear or any other emotion related to the situation, its persuasion, and the coping process. This theory directly indicates the motives of parents of applying mediations based on their perception about the threat (severity and susceptibility) and coping appraisal (response-efficacy and self-efficacy; Rogers, 1983; Norman et al., 2005; Plotnikoff and Trinh, 2010).

This theory facilitates to comprehend the preference of parents for applying various dimensions of parental internet mediation (active co-use or instructive, restrictive, monitoring, technical and active internet safety mediation, Laissez Faire, or unmediated), subject to their understanding about online risks and efficacy of children/teenagers in coping up with risks. Broadly, this theory predicts that those parents who perceive online risks as more severe and susceptible to their children or teenagers apply more and diverse mediation. Nevertheless, the parents who perceive their children/teenager as more capable of having sufficient response-efficacy and self-efficacy to prevent risks and perform optimal behavior online apply less mediation or remain unmediated. For that reason, we recommend that parental internet mediation could be predicted as a protective behavior in the face of online risks. Considering the risk perception and protective actions, the protection motivation theory drives in understanding the online safety behavior, keeping in view the cultural variation (Menard et al., 2018). This theory facilitates understanding whether individual sociocultural values motivate to perform a protective behavior and adopt various dimensions of parental mediation.

**Figure 2:**
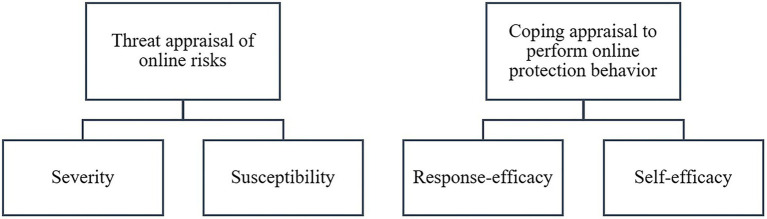
Protection motivation as a key predictor of parental internet mediation.

Derived from the protection motivation theory, Figure 2 elaborates the potential predictors or motivating factors of parental internet mediation, giving importance to both threat and coping appraisal at parental level, where threat appraisal indicates severity and susceptibility of online risks while coping appraisal signifies response and self-efficacy to prevent online risks.

### Theory of Resilience

Finally, this study introduces the concept of resilience for theoretical integration, which has various interpretations. Resilience is a dynamic concept and concerned with how individuals respond to risks, stress, or challenges (Rutter, 1987). Several researchers defined resilience in diverse ways, highlighting its compensatory, challenging, or protective factors, where resilience neutralizes exposure to risks, enhances the adaptation of person, or opted as an active problem-solving approach, fostering positive personality characteristics and wellbeing (Ledesma, 2014).

We argue that resilience is an effective measure to ensure the online safety of children/teens and to enhance online opportunities. Therefore, we adopted a socio-ecological approach to comprehend the key factors and process of resilience as potential predictors of parental internet mediation. Ungar (2011) theorized the social ecology of resilience as capacity of an individual, in the face of any challenge or adversity, to navigate and negotiate for resources in a culturally meaningful way to sustain wellbeing. In general, the theory of Ungar (2011) explained some basic principles of socio-ecology and emphasized the significance of resources and capacities to adopt resilient pathways. For instance, resilience is linked with own positive adjustments of children/teenagers against challenges or risks (herein referred to as online risks within the context of the internet), demonstrating their capacities to reciprocate and navigate for resources. Ungar (2011) has drawn attention to the role of family and friends as a valuable resource, providing necessary support to the children and teenagers in the face of any risk or challenge.

Realizing the connotation of resources in nurturing resilience among children and teenagers, it is meaningful to include these aspects in exploring the key factors of parental internet mediation, which vary according to diverse contexts and cultures. The literature revealed both internal and external level factors, which contribute to the ability of an individual to thrive (Carver, 1998; Ledesma, 2014). Internal factors are related to self and personality of an individual, having a significant impact on interpretation of an individual and dealing with adversity, such as positive attitude toward self, cognitive skills, constructive emotions and energy, self-regulation, self-efficacy, core personal values, and motivation to be effective in the environment (Luthar et al., 2000; Wyman et al., 2000; Bonanno, 2004; Patterson and Kelleher, 2005). Predominantly, it is argued that individuals with higher levels of positive personality traits and lower levels of disruptive behaviors are more likely to cope better with risks and build resilience (Affleck and Tennen, 1996; Park et al., 1996; Tedeschi and Calhoun, 1996). On the other hand, external factors influence the ability of an individual to remain resilient while facing adversity. For example, a strong support system and close relationships with family, friends, and community, as the most critical social resource, encourages and reinforces coping skills among individuals to adopt resilient pathways (Rutter, 1987; O’Leary, 1998; Masten, 2001). In this study, the internal and external factors highlight that resilience is indicative of both psychological and behavioral characteristics of children and teenagers.

Given the context of online digital environment of children/teenagers and the role of parental internet mediation, it may be stated that children and teenagers are at the exposure to unforeseen online risks. Therefore, parents applied multiple dimensions of parental mediation to build critical thinking, leading to the journey of resilience to reduce any potential harm. Theoretical analysis suggests that the possible predictors for nurturing resilience among children and teenagers could be personal skills, psychological and behavioral characteristics (higher levels of positive personality traits and lower levels of disruptive behaviors), and the support of parents, friends, and teachers to achieve resilient pathways. This study argues that resilience is a strength-based outcome and positive adjustment among children and teenagers against challenges or risks in the context of digital media. Resilience is not a linear process, rather it is iterative, related to the learning of children/teens on how to recognize and manage risks, learn from difficult experiences, and seek appropriate support to recover.

**Figure 3:**
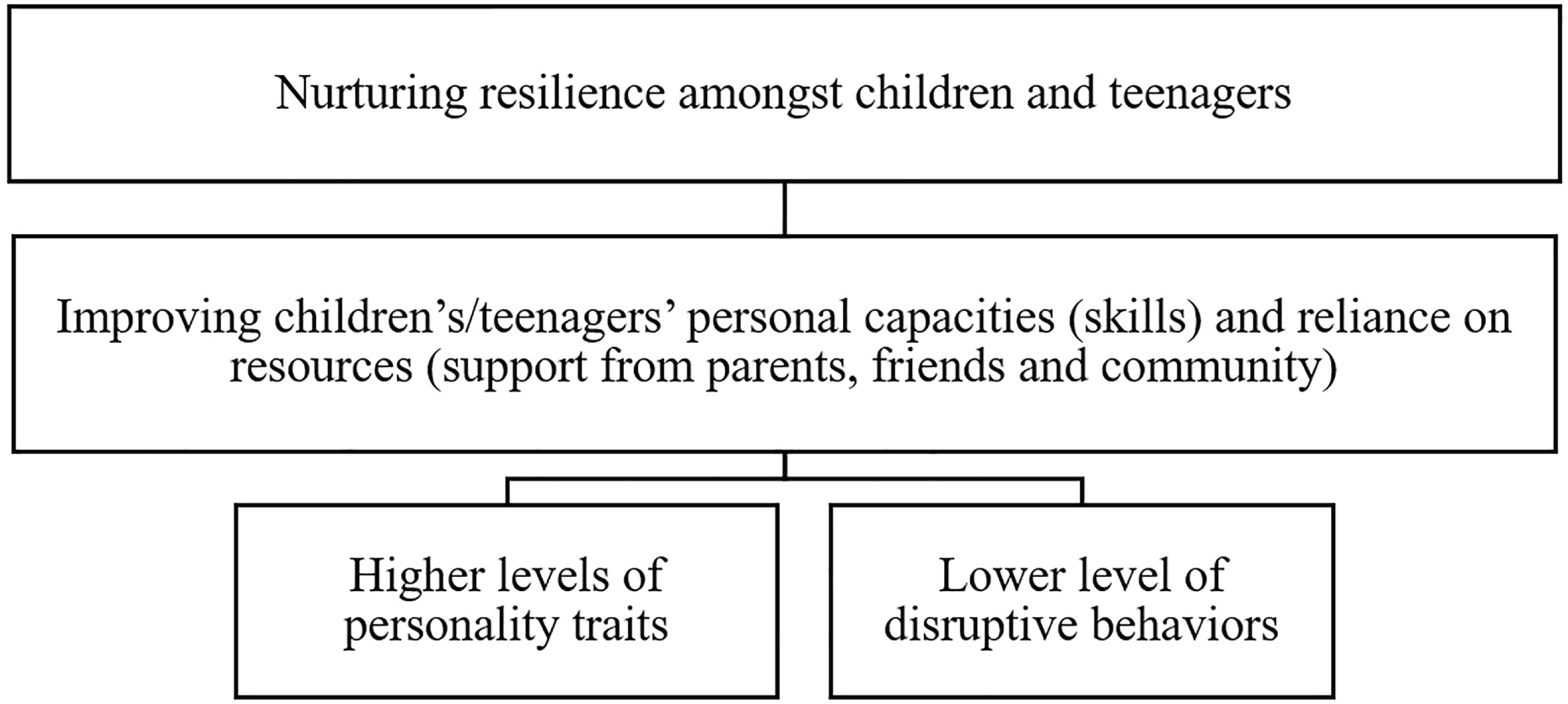
Factors and process of resilience as predictors of parental internet mediation.

Figure 3 depicts an illustrative explanation of nurturing resilience among children and teenagers, recognizing both psychological (e.g., personality traits) and behavioral predictors (e.g., disruptive behaviors) for an extended socio-ecological framework of parental internet mediation.

## Extended Theoretical Framework For Predictors of Parental Internet Mediation

In the light of the theories described above, we conclude that an integrated approach is critical to formulate the basis for a socio-ecological theoretical framework to fully comprehend the predictors and motivating factors of parental internet mediation. Broadly, this extended framework adopts the socio-ecological resilience model (Ungar, 2011), where children and teenagers are positioned within their environment along with their related characteristics, recognizing the interaction of predictors of multiple levels, particularly at parental and contextual levels. Taking inspiration from all the above given theories, this study also represents resilience as a strength-based discourse to promote positive outcomes among children and teenagers to manage online difficulties. This study acknowledges resilience as both a process and positive outcome against online risks and challenges, resulting from parental internet mediation. Resilience is an iterative process, related to the learning of children/teens, enabling them to recognize and manage online risks, learn from difficult experiences, and seek appropriate support to recover. It argues that resilience is an effective measure to ensure the online safety of children/teens and to enhance online opportunities.

**Figure 4:**
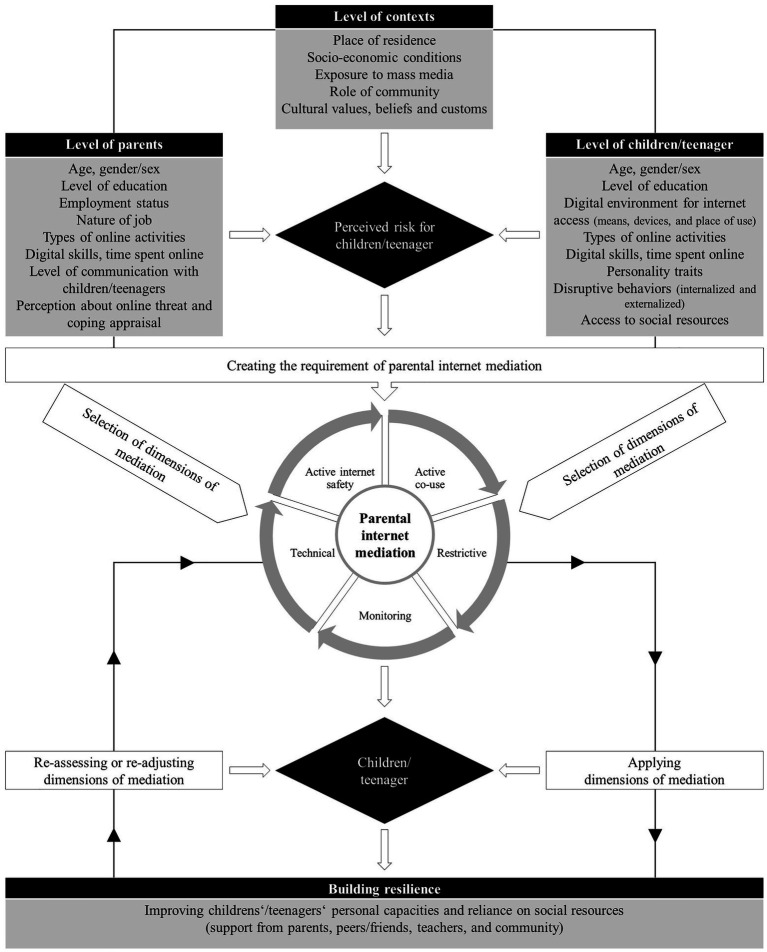
Proposed theoretical framework for the predictors of parental internet mediation.

Seeking guidance from the above theories, this study applies a socio-ecological and multi-methodological approach, unveiling a set of essential predictors to be considered at the level of parents, children/teenagers, and contexts. First, in the level of children/teenagers, the individual characteristics are the most significant, such as age, gender/sex, level of education, digital environment for internet access (means, devices, and place of use), types of online activities, digital skills, time spent online, positive personality traits, less disruptive behaviors, and access to social resources, in the form of support from parents, friends/peers, and teachers for building resilience. Second, in the level of parents, their particular characteristics and beliefs are essential for empirical investigation, e.g., the level of education of parents, employment status, nature of job and workplace, income, own use of the internet, digital skills, the level of communication with children/teenagers, threat appraisal about online risks (severity and susceptibility), and coping appraisal to perform optimal protection behaviors (response-efficacy and self-efficacy). Third, contextual level factors are imperative to be investigated, such as place of residence, socio-economic conditions of families, and cultural values, beliefs, and customs. Figure 4 illustrates the proposed socio-ecological extended framework through connecting the dots and creating linkages among multiple factors of parental internet mediation.

Based on the above synthesis and prepositions, the proposed extended socio-ecological model combines multiple individuals, contextual, behavioral, and analytical predictors, altogether from both the levels of children/teenagers and parents. We argue that it is necessary to apply a socio-ecological approach to investigate the potential predictors for parental internet mediation, particularly nurturing resilience among children/teenagers to manage online risks in the digital context of contemporary societies.

This extended framework model has also been empirically tested in primary research using both quantitative and qualitative designs (Iqbal, 2019; Iqbal et al., 2021). Previous evidence suggests that the above-given set of socio-ecological predictors for the extended theoretical framework of parental internet mediation is quite comprehensive. Nonetheless, there is a requirement of unfolding certain factors to ensure its effective application, such as personality traits and disruptive behaviors, being psychological and behavioral predictors. The most common personality traits are studied in terms of the five big models (Goldberg, 1993), including extrovert vs. introvert, emotionally stable vs. neurotic, agreeable vs. disagreeable, conscientiousness vs. lack of direction, and openness vs. closeness to experiences. Among these, only the positive attributes of personality will help to build resilience. Conversely, the disruptive behavior, the negative emotions or reactions against a particular situation, could be internalized or externalized, focusing inward or outward (Achenbach et al., 1991). Regarding the internet, internalized disruptive behaviors may include high depression and anxiety or low self-esteem while externalized disruptive behaviors include high aggression, delinquency, or the use of drugs or smoking may also be considered. The operationalization of most of the predictors is available and is used in several studies (Achenbach et al., 1991; Goldberg, 1993; Livingstone and Helsper, 2008; Livingstone et al., 2015a; Iqbal, 2019; Iqbal et al., 2021).

Future research is also welcome to examine, validate, and test the relationship between the predictors of the proposed framework of parental internet mediation for a more in-depth understanding. Further, researchers may also extend the proposed socio-ecological framework, integrating innovative approaches and related predictors.

## Conclusion

Parents remain highly concerned of online behavior of children and teenagers for multiple reasons and apply mixed or diverse dimensions of internet mediation, i.e., active co-use, restrictive, monitoring, technical, internet safety mediation, or no mediation. The preference of parents for selecting multiple dimensions of mediation or no mediation is subject to their socio-economic conditions (age, education, employment status, job nature, and income), own use of the internet (time spent online and types of online activities), digital skills, and the level of communication with children/teenagers (positive, neutral, and negative). Further, the perception of parents related to threat appraisal of children/teenagers about online risks (severity and susceptibility) as well as the perception about coping appraisal of children/teenagers to perform optimal protection behaviors (response-efficacy and self-efficacy) also determine their preference for parental internet mediation. Parental internet mediation facilitates building critical thinking and nurturing resilience among children and teenagers to manage online risks and ensure online safety. Therefore, it is a prerequisite to explore the positive personality traits of children and teenagers (extrovert, emotionally stable, agreeable, conscientiousness, and openness to experiences), less disruptive behaviors (depression or anxiety, self-esteem, aggression, delinquency, or the use of drugs/smoke), and access to social resources (the support from parents, peers/friends, teachers, and community). The socio-ecological approach also suggests in considering the contextual factors of parental internet mediation, e.g., place of residence, socio-economic conditions of families, and cultural values, beliefs, and customs. A graphical illustration of the potential socio-ecological predictors is given in Figure 4.

To sum up, this contribution argues that parental internet mediation is an ongoing process, where parents reassess and readjust their dimensions of internet mediation based on the feedback and reaction of children and teenagers with an ultimate intention to build a strength-based outcome of resilience among them. Therefore, this theoretical review is an attempt to enrich the sociological inquiry of parental internet mediation using a multi-grounded theoretical approach. We want to enrich the academic discourse and reflect on the varied theoretical stances to explore the potential predictors of parental internet mediation. The proposed extended theoretical framework facilitates to provide an insight to comprehend the predictors of parental internet mediation more concisely, at the levels of parents, children/teenagers, and contexts, particularly highlighting resilience as a positive adjustment among children and teenagers against challenges or online risks in the context of digital media. This analytical perspective considers both psychological and behavioral predictors of parental internet mediation to adopt a resilient pathway.

An essential contribution of this study is related to its objective, acknowledging the need for an overarching framework of parental internet mediation in the context of a digital media-rich environment. Subsequently, this study presents an extended and a comprehensive socio-ecological framework for parental internet mediation. Future research is needed to empirically test this extended model in various settings.

## Author Contributions

SI: conceptualization, investigation, and writing – original draft preparation. RZ and FF: supervision and writing – review and editing. All authors contributed to the article and approved the submitted version.

### Conflict of Interest

The authors declare that the research was conducted in the absence of any commercial or financial relationships that could be construed as a potential conflict of interest.
